# Differences in Diagnosing Tarsal Tunnel Syndrome Across the Literature: A Systematic Review and a Call for Standardization

**DOI:** 10.2106/JBJS.RVW.25.00222

**Published:** 2026-02-09

**Authors:** Nadine Boers, Melanie Haverkamp, Anne Merijn Eligh, Manuel Castro Cabezas, J. Henk Coert, Willem D. Rinkel

**Affiliations:** 1 Department of Plastic, Reconstructive, and Hand Surgery, Utrecht University Medical Center, Utrecht, the Netherlands; 2 Department of Internal Medicine, Center for Diabetes and Vascular Medicine, Sint Franciscus Gasthuis, Rotterdam, the Netherlands; 3 Department of Plastic, Reconstructive, Hand, and Wrist Surgery, ZGT/MST, Twente, the Netherlands

## Abstract

**Background:**

The lack of a gold standard in tarsal tunnel syndrome (TTS) diagnosis leads to diagnostic inconsistencies and variation in patient selection for treatment. Therefore, the aim of this review is to summarize the diagnostic criteria used in current studies on TTS based upon this best-evidence synthesis.

**Methods:**

Three databases were searched to identify all studies on TTS. Studies were included when they included (1) diagnosis or treatment of TTS as the primary focus, (2) a description of the diagnosis of TTS, (3) an original data set of TTS cases, and (4) a minimum of 10 adult patients diagnosed with TTS. A best-evidence synthesis was used to summarize the results.

**Results:**

In total, 4,213 patients were represented in 82 included studies. Among the varying diagnostic methods employed, aside from clinical symptoms, provocative testing was most often used (in 94% of studies, mandatory for diagnosis in 41% of studies) with the Tinel sign being the most prevalent (used in 89% of studies). Sensitivities of provocative tests, electrodiagnostic, and ultrasound measurements showed significant variability.

**Conclusion:**

We provided an overview of the diagnostic tools and workups reported in the literature on TTS. Our findings show that the lack of a standardized diagnostic approach results in considerable variability in clinical practice. Alongside typical clinical symptoms, the Tinel sign is the most frequently used diagnostic test. The varying sensitivities reported in literature underscore the need for evidence-based diagnostic guidelines on TTS diagnosis.

**Level of Evidence:**

Diagnostic Level III. See Instructions for Authors for a complete description of levels of evidence.

The first description of tibial nerve entrapment at the tarsal tunnel dates from 1918^1^. Keck and Lam described tarsal tunnel syndrome (TTS) independently in 1962^2,3^. Since then, multiple studies have been published on clinical symptoms, physical examination, electrodiagnostic studies, imaging, and diagnostic tools. However, there is still controversy in which diagnostic modalities contribute to the clinical diagnosis and a gold standard does not exist. Consequently, patient selection for different treatment options varies between practices^4^.

TTS can present as a mononeuropathy, but also as a superimposed compression neuropathy, particularly in patients with diabetes and diabetic sensorimotor polyneuropathy (DSP). In recent decades, more results have been reported on the effects of decompression of the tibial nerve at the tarsal tunnel on outcomes like pain, neuropathic symptoms and diabetic foot ulcer occurrence in this patient group. The presence of superimposed compression neuropathies, including TTS in patients with DSP, is a reason for optimism in this vulnerable group, since treatment options become available when the diagnosis is made^5^. However, clinical decision making remains difficult, because of similarities in clinical symptoms with other neuropathies based on a shared pathophysiology (i.e., demyelinization, axonal loss/sprouting), which is echoed in, for example, nerve conduction parameters^6,7^.

Therefore, the aim of this review was to summarize the literature-reported diagnostic modalities used in studies evaluating the diagnosis, prevalence, and/or treatment of TTS. This best-evidence synthesis may further aid epidemiologic characterization of this entity.

## Methods

### Protocol and Registration

This review was performed according to the Preferred Reporting Items for Systematic Review and Meta-Analysis for Diagnostic Test Accuracy guideline^8^. The review protocol has not been registered before.

### Search Strategy

A literature review was conducted to identify all studies on TTS. PubMed, Embase, and Cochrane Library were searched up to August 2023. The following key terms and Medical Subject Headings descriptors or Emtree terms were used: “tarsal tunnel,” “tibial neuropathy,” and “tibial nerve entrapment.” The complete search strategy can be found in Supplementary File A.

### Study Selection

Identified studies were included if they met prespecified eligibility criteria: (1) the primary focus of the article is diagnosis or treatment of TTS; (2) a description of the diagnosis of TTS in the selected study population is given; (3) an original data set of TTS cases was used; (4) the study population consisted of a minimum of 10 adult patients diagnosed with TTS. Exclusion criteria were (1) cadaver or animal studies and (2) languages other than English or Dutch. Screening of titles, abstracts, and subsequently full text was performed by 2 independent researchers (M.H. and N.B.). A third reviewer (W.D.R.) was consulted in case of discrepancies in selected studies and a consensus method was used to solve any disagreements. In addition, references of included studies were reviewed for additional studies.

### Data Extraction

Two authors (M.H. and N.B.) independently extracted data from the included articles. Information was collected on the publication year, author, study population, sample size, etiology of TTS, diagnostic modalities used, whether these modalities were mandatory for diagnosis, the number of patients in whom a nonmandatory modality was positive, study aim, and exclusion criteria.

### Risk of Bias Assessment

Two reviewers (A.M.E., N.B.) independently assessed the methodological quality of studies reporting diagnostic accuracy, using the QUADAS-2 tool for diagnostic studies^9^. Disagreements were resolved by consensus, with a third reviewer (W.D.R.) consulted if needed. Overall risk of bias was defined as: low (low risk in all 4 domains), some (high risk in 1 domain, with or without unclear risk in 1 domain), or high (high risk in more than 1 domain, or high risk in 1 domain plus unclear risk in 2 or more domains, or unclear risk in 3 or more domains). QUADAS-2 results are shown in tables and graphs for each domain in Supplementary File A.

### Diagnostic Accuracy Measures

Sensitivity of diagnostic modalities was extracted or calculated when possible, using the formula: true positives/(true positives + false negatives). Diagnostic accuracy was based on the number of feet to account for bilateral disease.

### Data Synthesis

A quantitative analysis of the studies was not feasible because of the heterogeneity in outcome measures. Instead, we applied a best-evidence synthesis^10^. This combines the principles of systematic review and narrative synthesis by qualitatively summarizing the highest-quality available studies when quantitative meta-analysis is not feasible. It weighs evidence strength based on study design, methodological quality, and consistency of findings to provide the most reliable overall conclusion.

Studies were included in this synthesis only if sensitivity was reported or could be derived from the data. Sensitivity was calculated from the number of positive results (using the article's own positivity thresholds and reference standards, which varied) and the number of TTS patients. Diagnostic modalities used solely to evaluate treatment efficacy, rather than to diagnose TTS, were excluded. The median, range, and interquartile range (IQR) of sensitivities were calculated for each diagnostic modality. Missing values were not imputed. All statistical analyses were performed using IBM SPSS version 26.0.

## Results

### Study Characteristics

**Fig. 1:**
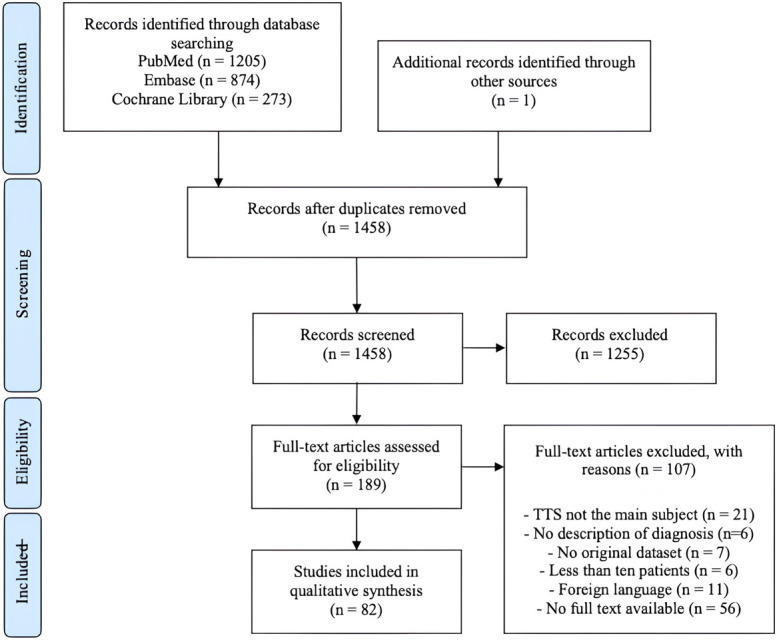
PRISMA flow diagram. PRISMA = Preferred Reporting Items for Systematic Review and Meta-Analysis, and TTS = tarsal tunnel syndrome.

A total of 1,458 unique studies were identified and were screened based on title and abstract (Fig. 1). Eighty-two studies were included, encompassing 4,213 patients with 4,509 feet with TTS^11-50,51–80,81-93^. Details on study characteristics for individual studies are shown in Supplementary File B.

### Etiology of TTS

**Fig. 2:**
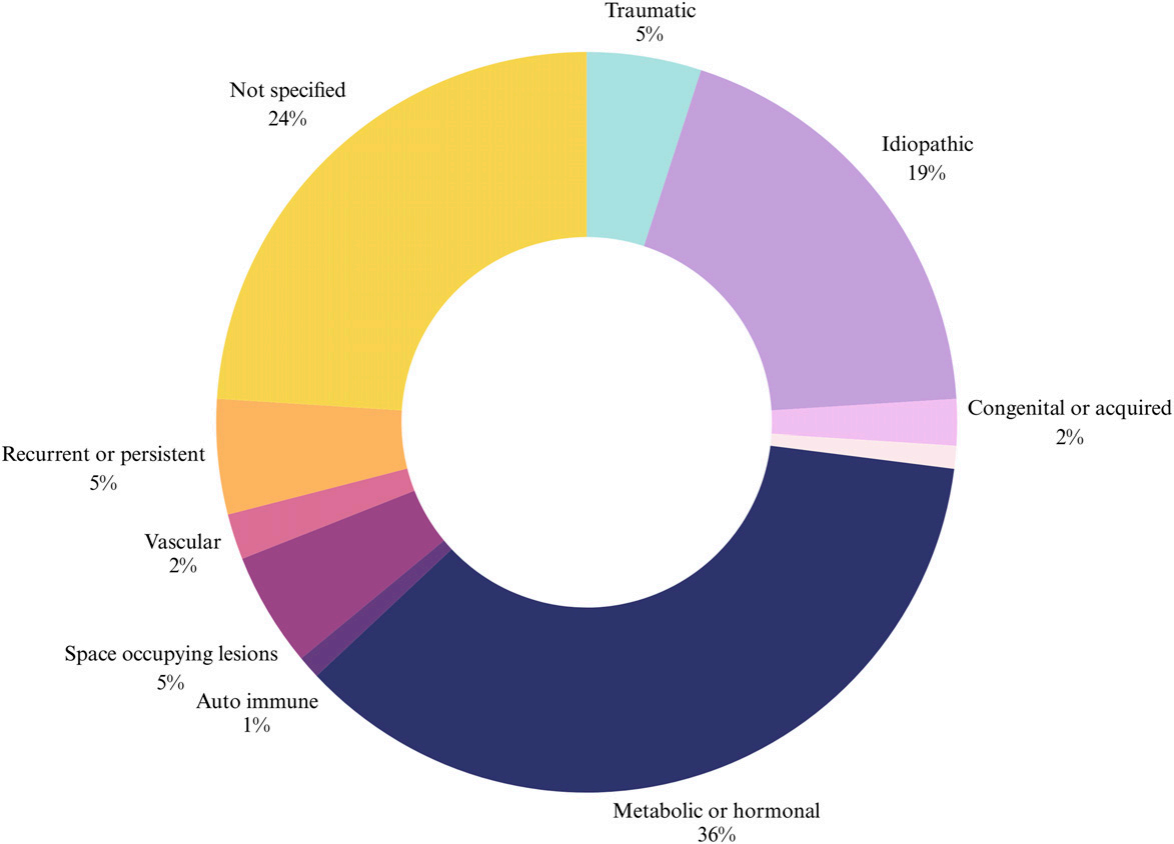
Etiology of TTS as reported in the literature. TTS = tarsal tunnel syndrome.

Etiology of TTS was reported in 76% of patients, as shown in Figure 2. Reported underlying pathologies included diabetes mellitus (30% of patients), diabetes with DSP (23%), pregnancy (4%), rheumatoid arthritis (1%), and fibromyalgia (<1%).

### Diagnostic Modalities

**Fig. 3:**
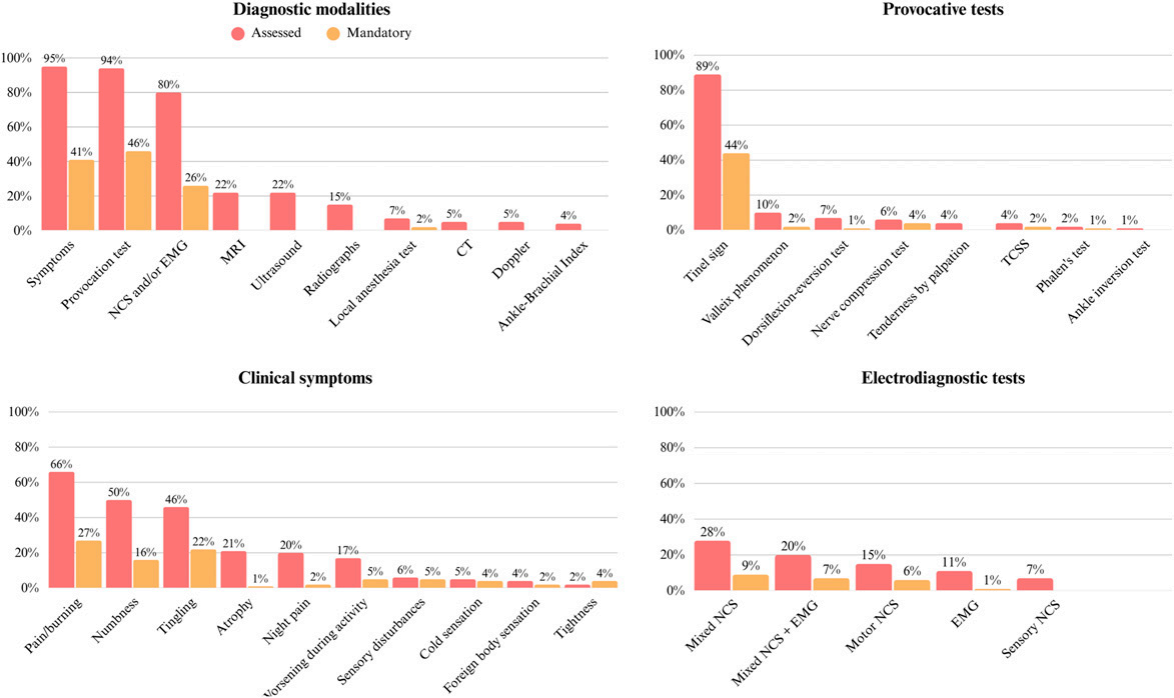
Diagnostic modalities, clinical symptoms and provocative tests used for TTS diagnosis. The Y axis represents the percentage of studies. CT = computed tomography, EMG = electromyography, MRI = magnetic resonance imaging, NCS = nerve conduction studies, TCSS = triple compression stress test, and TTS = tarsal tunnel syndrome.

Table I presents the diagnostic modalities mandatory, and their combinations, reported in the literature as mandatory for TTS diagnosis. Figure 3 presents the percentage of studies using each diagnostic modality and the proportion requiring a positive result for TTS diagnosis.

**TABLE I:** Diagnostic Criteria Mandatory for TTS Diagnosis

Diagnostic Modalities	N Studies (%)
Clinical symptoms only	25 (30)
Positive electrodiagnostic studies only	3 (4)
Positive provocative test only	1 (1)
Clinical symptoms *or* positive provocative test	1 (1)
Clinical symptoms *or* positive electrodiagnostic studies	1 (1)
Positive provocation test *or* positive electrodiagnostic studies	1 (1)
Clinical symptoms *and* positive provocative test	25 (30)
Clinical symptoms *and* positive electrodiagnostic studies	8 (10)
Positive provocation test *and* positive electrodiagnostic studies	4 (5)
Clinical symptoms *and* positive provocative test *and* positive electrodiagnostic studies	7 (9)
Not specified	6 (7)

TTS = tarsal tunnel syndrome.

#### Diagnostic Accuracy

Sensitivities were extracted from 52 studies^11,14-16,21,27,31-34,38,44,45,47-54,56,58,59,60-63,65,66,68,69,71-79,82-85,87-92^. The median, range, and IQR were of sensitivities for each diagnostic modality are described in Table II, with a comprehensive list in Supplementary File B.

**TABLE II:** Reported Diagnostic Accuracy of Diagnostic Tests in Patients Diagnosed with TTS

	N Studies	Median Sensitivity (IQR)	Range	Overall Risk of Bias, N (%)		
				Low	Some	High
Provocative test						
Tinel sign	28	78.5 (36)	1, 100	—	19 (68)	9 (32)
Dorsiflexion-eversion test	4	62 (16)	29, 86	—	3 (75)	1 (25)
Valleix phenomen	4	43 (38.5)	4, 61	—	3 (75)	1 (25)
Nerve compression test	3	79 (27)	46, 100	—	1 (33)	2 (67)
Local tenderness behind the MM	1	98	—	—	—	1 (100)
Triple compression test	1	86	—	—	—	1 (100)
Ankle inversion test	1	32	—	—	1 (100)	—
Electrodiagnostic studies						
Mixed NCS	14	66 (40)	21, 100	—	7 (50)	7 (50)
Sensory NCS	15	84 (15)	23, 100	—	5 (33)	10 (67)
Motor NCS	15	57 (37)	21, 91	—	5 (33)	10 (67)
EMG	7	86 (43)	0, 93	—	2 (29)	5 (71)
Mixed NCS + EMG	6	78.5 (12)	34, 97	—	3 (50)	3 (50)
Imaging studies						
CSA measurement	3	61 (19)	36, 100	1 (25)	2 (50)	1 (25)
CSA-ratio	1	74	—	1 (100)	—	—
MR nerve hyperintensity	1	62	—	—	—	1 (100)
Other diagnostic tools						
Local anesthetic injection	2	72 (18)	64, 100	—	2 (67)	1 (33)

CSA = cross-sectional area, EMG = electromyography, IQR = interquartile range, MM = medial malleolus, MR = magnetic resonance, NCS = nerve conduction study, and TTS = tarsal tunnel syndrome.

Gold standard used to assess sensitivity varied between studies.

#### Risk of Bias

**Fig. 4:**
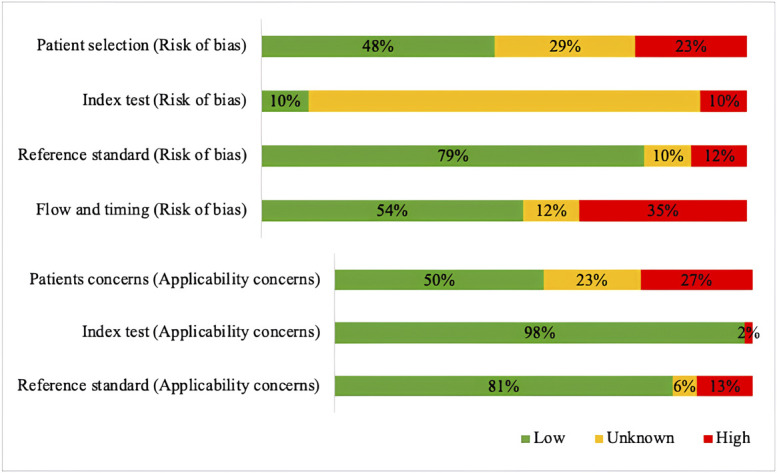
Risk of bias graph.

The overall risk of bias was low in 1 study (2%), some risk of bias was found in 30 studies (59%), and a high risk of bias was noted in 20 studies (39%). High risk of bias was mainly due to the unblinded nature of most studies and the absence of appropriate exclusion criteria. Figure 4 shows the risk of bias for each domain and concerns regarding applicability. Outcomes for each study separately are shown in Supplementary File A.

#### Clinical Symptoms

Clinical symptoms were used in 95% (78/82) of the studies and the presence of symptoms was reported to be mandatory for TTS diagnosis in 41% (34/82) of the studies. Predominantly, these symptoms included pain or a burning sensation (66%, 54/82), numbness (50%, 41/82), and/or tingling (46%, 38/82) in the distribution area of the tibial nerve.

#### Provocative Tests

Provocative tests were used in 94% (77/82) of the studies and reported to be mandatory for TTS diagnosis in 41% (34/82). In total, 8 different provocative tests were used. Each provocative test is described in detail in Table III. The Tinel sign at the tarsal tunnel was the most used, in 89% (73/82) of studies, followed by the Valleix phenomenon at the ankle (10%, 8/82), the dorsiflexion-eversion test (7%, 6/82) and the nerve compression test at the tarsal tunnel (6%, 4/82). Twenty-eight studies reported diagnostic accuracy (median 78.5, IQR 36) as shown in Table II, with 19 studies having some risk of bias and 9 studies having a high risk of bias.

**TABLE III:** Provocative Tests Used for TTS Diagnosis

Provocative Test	
Tinel sign	Eliciting or increasing a sensation of tingling or “pins and needles” in the distribution of the nerve by tapping on the tibial nerve in the tarsal tunnel
Nerve compression test	Eliciting or increasing symptoms by performing pressure over the tarsal tunnel for over 15 seconds eliciting symptoms
Valleix phenomenon	Eliciting or increasing pain radiating proximal to the nerve course by percussion of the nerve at the ankle
Phalen's test	Eliciting or increasing symptoms when passively, maximally everting the ankle and holding the ankle in this position
Dorsiflexion-eversion test	Eliciting or increasing symptoms by placing the patient's foot into dorsiflexion and eversion for 15 seconds while maintaining extension of the metatarsophalangeal joints
Ankle inversion test	Eliciting or increasing symptoms by placing the ankle in full plantar flexion with the foot in inversion
Triple compression stress test	Eliciting or increasing symptoms by applying pressure on the tibial nerve behind the medial malleolus for 30 seconds by the examiner's hand with the ankle in full plantar flexion with the foot in inversion
Palpation of the tibial nerve	Eliciting or increasing tenderness of the tibial nerve by palpation

TTS = tarsal tunnel syndrome.

#### Electrodiagnostic Studies

Electrodiagnostic studies, including nerve conduction studies (NCSs) and electromyography (EMG), were used in 80% (66/82) of the studies and abnormalities were reported to be mandatory for TTS diagnosis in 46% (38/82). Mixed NCS was most used (28%, 23/82), followed by mixed NCS combined with EMG (20%, 16/82), motor NCS (15%, 12/82), EMG alone (11%, 9/82), and sensory NCS (7%, 6/82). EMG had the highest median sensitivity (86, IQR 43), followed by sensory NCS (84, IQR 15). Studies used different reference values. Of the 35 studies reporting diagnostic accuracy of electrodiagnostic studies, 18 had some risk of bias and 17 a high risk of bias.

#### Imaging Techniques

Imaging techniques like ultrasound, magnetic resonance imaging, high-resolution magnetic resonance neurography, computed tomography scans, and Doppler and Ankle-Brachial Index were employed to determine etiology or exclude other diagnoses. Cross-sectional area (CSA) measurements, to detect an increased tibial nerve size, were assessed in 5 studies but were not deemed mandatory for diagnosis^11,25,56,84,90^. Of the 4 studies reporting diagnostic accuracy, the risk of bias varied from low to high.

#### Somatosensory Testing

Loss of somatosensory functions were evaluated in 13 studies (16%). Seven studies used monofilaments^30,80,90,91^ of which 1 study described a threshold of 10 g^91^, 1 study used it in a descriptive manner without a threshold^80^ and 2 studies used an unspecified threshold. Three studies used pinprick touch with an unspecified threshold^63,65,75^. Five studies assessed 2-point discrimination^22,30,31,45,81^ of which 1 study used it in a descriptive manner without a threshold^22^, 2 studies used an age-dependent threshold^30,31^, 1 study used an unspecified threshold^81^, and 1 study compared the outcomes with the unaffected feet^45^. Four studies used vibration sense of which 1 study compared the outcomes with the unaffected feet^45^ and other studies did not describe thresholds^31,48,80^.

#### Other Diagnostic Tests

A diagnostic block using injection of local anesthesia^33,51,68,77^ or corticosteroids^54^ was used in 5 studies (6%) of which 2 studies described a significant reduction or complete cessation of pain to be indicative for nerve compression^33,51^. Three studies did not specify how injection was performed and when it was deemed positive. One study considered a positive outcome necessary for TTS diagnosis^68^ and 1 study considered a positive outcome necessary for surgical treatment^51^.

## Discussion

In this systematic review, we summarized the literature on diagnostic criteria for TTS. The reviewed studies reported varying combinations of clinical symptoms, provocative tests, and electrodiagnostic studies used for TTS diagnosis. We found substantial variability in reported sensitivities, largely due to the absence of a robust gold standard for diagnosing TTS across diverse patient populations—from otherwise healthy individuals with idiopathic TTS to patients with diabetic neuropathy and superimposed TTS. Overall, the evidence synthesis indicates a lack of consensus and a low level of evidence supporting the diagnostic criteria currently in use, highlighting the need for consensus on diagnosis of TTS.

Thirty-five percent of studies used only a single indicative modality for TTS diagnosis, which may increase the risk of false positives. Ideally, diagnosis combines clinical symptoms with at least 1 diagnostic criterion, which is used in 40% of included studies with varying combinations. Twenty-three percent of studies (19/82) combined multiple provocative tests^13,16,18,27,32,33,35,38,40,41,45,50,77,79,85,86,88-90^ and in 5 studies 2 or 3 positive provocative tests were required for diagnosis^13,18,35,40,86^. However, using multiple tests with variable sensitivities may increase the risk of false negatives. Unfortunately, receiver operating characteristic (ROC) curves comparing the accuracy of single versus multiple diagnostic tools have not been reported.

Provocative tests were the most often advocated and studied diagnostic maneuver and are employed in 89% of reviewed studies. Of these, the Tinel sign was the most used. The wide range of sensitivities reported (ranging from 1% to 100%) may be explained by the chosen gold standard comparator and by the pathophysiology of chronic nerve compression. As theorized by Dellon^94^, the Tinel sign is thought to be negative at the earliest stage of compression but becomes positive with longer duration or degree of compression, resulting in demyelinization of axons. With continued compression, axons degeneration occurs, yet the Tinel sign may remain positive even in advanced stages due to remyelination and axonal regeneration. If the nerve fails to recover from the damage, the Tinel sign will eventually become negative. This pathophysiological background should be considered when interpreting both the chosen gold standard and the Tinel sign.

The second most frequently used diagnostic modality was electrodiagnostic testing, most often mixed (sensory and motor) NCS, though the specific tests varied. Reported sensitivities ranged widely, likely influenced by procedural factors (e.g., room and foot temperature, electrode distance) and patient characteristics (e.g., age, foot size, and presence of edema). In addition, diagnostic thresholds differed across studies, and some did not report their methods or thresholds at all, making it impossible to appropriately compare or draw conclusion from the reported sensitivities.

CSA measurements were less frequently used. This modality is noninvasive and less time-consuming compared with electrodiagnostic testing. However, only 3 studies of varying quality (low to high) reported sensitivity, each using different reference standards. Given the small number of studies, estimates may be inflated due to publication bias^11,25,56,84,90^. Only 1 study assessed both CSA cutoff values and CSA ratios using ROC curve analysis, finding the within tunnel-to-proximal tunnel CSA ratio to be the most sensitive parameter (74%), followed by within tunnel CSA (61%)^84^. However, more studies are needed to validate the diagnostic value of CSA measurements in TSS.

Diagnosing TTS is challenging due to overlap with other lower extremity disorders, particularly in patients with diabetes and DSP, where symptoms of TTS can be increased due to the existing neuropathic symptoms^80^. The diffuse damage to peripheral nerves (first crush) makes it difficult to distinguish entrapment related demyelinization/axonal loss (second crush). Diagnostic results should therefore be interpreted with caution, considering preexisting diabetic neuropathy and nerve damage by diabetes. To date, no studies have reported the diagnostic accuracy of electrodiagnostic testing or ultrasound for TTS in patients with DSP.

Studies looking into carpal tunnel syndrome (CTS) in DSP illustrate these challenges. Heiling et al. found that in patients with DSP, neither electrodiagnostic testing, median nerve CSA, or the wrist-to-forearm ratio distinguished between patients with and without CTS symptoms^95^. Perkins et al. likewise reported that electrodiagnostic testing could not detect superimposed CTS in diabetic patients with DSP^96^. In a large diabetic cohort, the Tinel sign was observed across the spectrum of neuropathy, from no sensory deficit to end-stage sensory loss^97^. A follow-up study in the same cohort suggested the Tinel sign may still be valuable for diagnosing TTS in DSP, as patients with a positive Tinel sign showed higher cutaneous thresholds and more neuropathic symptoms^80^.

Current treatment options for TTS typically start conservative, including rest, physical therapy, massage, taping, stretching, and medications aimed at alleviating neuropathic pain^98^. If these fail, or a structural cause is identified, surgical decompression may be performed. When considering a diagnostic workup, not only diagnostic accuracy but also the ability to predict surgical outcome is relevant. Three studies found the Tinel sign to be a reliable indicator of symptom improvement after decompression^24,68,94^, whereas 2 studies reported that positive provocative tests did not predict better outcomes^58,77^. Evidence for electrodiagnostic testing is similarly inconsistent: 5 studies concluded that nerve conduction studies were not predictive of surgical success^31,58,60,68,83^, while 1 study reported them to be a good predictor of patient-reported improvement^77^.

Our review has several important limitations. It is constrained by incomplete data reporting, the absence of proper control groups, and a lack of well-conducted diagnostic accuracy studies. Consequently, we could not calculate true mean sensitivity, specificity, or predictive values. Although we summarized the wide range of reported sensitivities, these cannot be directly compared, precluding firm conclusions. Our quality assessment identified only 1 study with low risk of bias, while most showed some or high risk. Thus, our best-evidence synthesis should be interpreted with caution, as reporting bias may lead to overestimation or underestimation of sensitivity, with extreme values being more likely to be published.

## Conclusion

Our findings show that the lack of a standardized diagnostic approach results in considerable variability in clinical practice. Alongside typical clinical symptoms, the Tinel sign is the most frequently used diagnostic test. This review highlights the urgent need for evidence-based diagnostic and treatment guidelines and underscores the importance of large prospective cohort studies to establish standardized criteria for TTS diagnosis. The Delphi method could further aid that process to identify the best possible next step.

### Sources of Funding

No funding was provided in the investigation of this study.

## Appendix

Supporting material provided by the authors is posted with the online version of this article as a data supplement at jbjs.org (http://links.lww.com/JBJSREV/B308). This content was not copyedited or verified by JBJS.
